# Supplementary material for: Evaluation of the discriminatory power of spoligotyping and 19-locus mycobacterial interspersed repetitive unit-variable number of tandem repeat analysis (MIRU-VNTR) of Mycobacterium bovis strains isolated from cattle in Algeria
Source: PLoS One. 2022 Jan 11;17(1):e0262390. doi: 10.1371/journal.pone.0262390 (PMC8751994; doi:10.1371/journal.pone.0262390)
Supplement: Attachment — Submitted filename: PONE-D-21-26506.pdf [file pone.0262390.s002.pdf]

PLOS ONE

Evaluation of the discriminatory power of spoligotyping and 19-locus mycobacterial interspersed repetitive unit-variable number of tandem repeat analysis (MIRU-VNTR) of Mycobacterium bovis strains isolated from cattle in Algeria

--Manuscript Draft--

**Manuscript Number:**	PONE-D-21-26506
**Article Type:**	Research Article
**Full Title:**	Evaluation of the discriminatory power of spoligotyping and 19-locus mycobacterial interspersed repetitive unit-variable number of tandem repeat analysis (MIRU-VNTR) of Mycobacterium bovis strains isolated from cattle in Algeria
**Short Title:**	Mycobacterium bovis from cattle in Algeria
**Corresponding Author:**	Stefanie Barth<br>Friedrich-Loeffler-Institut Bundesforschungsinstitut fur Tiergesundheit<br>Jena, GERMANY
**Keywords:**	Mycobacterium bovis; Mycobacterium caprae; molecular characterization; spoligotyping; MIRU-VNTR-typing; cattle; Algeria
**Abstract:**	Bovine tuberculosis (bTB) caused by Mycobacterium (M.) bovis and M. caprae is a transmissible disease of livestock, notifiable to the World Organization for Animal Health (OIE). BTB particularly affects cattle and small ruminants and can be transmitted to humans thereby posing a significant threat to veterinary and public health worldwide. M. bovis is the principal cause of bTB in Algeria. In order to better understand the route of spreading and elaborate an eradication program, isolation and characterization of mycobacteria from Algerian cattle has been performed. Sixty strains belonging to the M. tuberculosis complex have been analyzed by spoligotyping, thereof 42 by 19-locus-MIRU-VNTR-typing. Spoligotyping revealed 16 distinguishable patterns (Hunter-Gaston discriminatory index [HGDI] of 0.8294), with types SB0120 (n = 20) and SB0121 (n = 13) being the most frequent patterns, representing 55% of the strains. Analyses based on 19-locus-MIRU-VNTR yielded 32 different profiles, five clusters and one orphan pattern, showing higher discriminatory power (HGDI = 0.9779) than spoligotyping. Seven VNTR-loci [VNTR 577 (alias ETR C), 2163b (QU11b), 2165 (ETR A), 2461 (ETR B), 3007 (MIRU 27), 2163a (QUB11a) and 3232 (QUB 3232)] were the most discriminative loci (HGDI ˃ 0.50). In conclusion, 19-locus-MIRU-VNTR yielded more information than spoligotyping concerning molecular differentiation of strains and better supports the elucidation of transmission routes of M. bovis between Algerian cattle herds.
**Order of Authors:**	Faïza Belakehal
	Stefanie A. Barth
	Christian Menge
	Hamdi T. Mossadak
	Naïm Malek
	Irmgard Moser
**Additional Information:**	
**Question**	**Response**
**Financial Disclosure**<br><br>Enter a financial disclosure statement that describes the sources of funding for the work included in this submission. Review the submission guidelines for detailed requirements. View published research	Parts of this work were funded by a travel and stay grant from the High National Veterinary School, Algeria, (ENSV, www.ensv.dz) (grant no. 000592) for Faiza Belakehal. The funders had no role in study design, data collection and analysis, decision to publish, or preparation of the manuscript.

Powered by Editorial Manager® and ProduXion Manager® from Aries Systems Corporation

articles from *PLOS ONE* for specific examples.<br><br>This statement is required for submission and **will appear in the published article** if the submission is accepted. Please make sure it is accurate.<br><br>**Unfunded studies**<br>Enter: *The author(s) received no specific funding for this work.*<br><br>**Funded studies**<br>Enter a statement with the following details:<br>• Initials of the authors who received each award<br>• Grant numbers awarded to each author<br>• The full name of each funder<br>• URL of each funder website<br>• Did the sponsors or funders play any role in the study design, data collection and analysis, decision to publish, or preparation of the manuscript?<br>• **NO** - Include this sentence at the end of your statement: *The funders had no role in study design, data collection and analysis, decision to publish, or preparation of the manuscript.*<br>• **YES** - Specify the role(s) played.<br><br>* typeset	
**Competing Interests**<br><br>Use the instructions below to enter a competing interest statement for this submission. On behalf of all authors, disclose any competing interests that could be perceived to bias this work—acknowledging all financial support and any other relevant financial or non-financial competing interests.<br><br>This statement is required for submission and **will appear in the published article** if the submission is accepted. Please make sure it is accurate and that any funding sources listed in your Funding Information later in the submission form are also declared in your Financial Disclosure statement.	The authors have declared that no competing interests exist.

View published research articles from *PLOS ONE* for specific examples.<br><br>**NO authors have competing interests**<br><br>Enter: *The authors have declared that no competing interests exist.*<br><br>**Authors with competing interests**<br><br>Enter competing interest details beginning with this statement:<br><br>*I have read the journal's policy and the authors of this manuscript have the following competing interests: [insert competing interests here]*<br><br>* typeset	
**Ethics Statement**<br><br>Enter an ethics statement for this submission. This statement is required if the study involved:<br><br>• Human participants<br>• Human specimens or tissue<br>• Vertebrate animals or cephalopods<br>• Vertebrate embryos or tissues<br>• Field research<br><br>Write "N/A" if the submission does not require an ethics statement.<br><br>General guidance is provided below. Consult the submission guidelines for detailed instructions. **Make sure that all information entered here is included in the Methods section of the manuscript.**	All samples were obtained at selected slaughterhouses in Algeria during routine meat inspection procedure. No additional permission was needed.

Powered by Editorial Manager® and ProduXion Manager® from Aries Systems Corporation

**Format for specific study types**<br><br>**Human Subject Research (involving human participants and/or tissue)**<br>• Give the name of the institutional review board or ethics committee that approved the study<br>• Include the approval number and/or a statement indicating approval of this research<br>• Indicate the form of consent obtained (written/oral) or the reason that consent was not obtained (e.g. the data were analyzed anonymously)<br><br>**Animal Research (involving vertebrate animals, embryos or tissues)**<br>• Provide the name of the Institutional Animal Care and Use Committee (IACUC) or other relevant ethics board that reviewed the study protocol, and indicate whether they approved this research or granted a formal waiver of ethical approval<br>• Include an approval number if one was obtained<br>• If the study involved *non-human primates*, add *additional details* about animal welfare and steps taken to ameliorate suffering<br>• If anesthesia, euthanasia, or any kind of animal sacrifice is part of the study, include briefly which substances and/or methods were applied<br><br>**Field Research**<br><br>Include the following details if this study involves the collection of plant, animal, or other materials from a natural setting:<br>• Field permit number<br>• Name of the institution or relevant body that granted permission	
**Data Availability**<br><br>Authors are required to make all data underlying the findings described fully available, without restriction, and from the time of publication. PLOS allows rare exceptions to address legal and ethical concerns. See the PLOS Data Policy and FAQ for detailed information.	Yes - all data are fully available without restriction

A Data Availability Statement describing where the data can be found is required at submission. Your answers to this question constitute the Data Availability Statement and **will be published in the article**, if accepted.<br><br>**Important:** Stating 'data available on request from the author' is not sufficient. If your data are only available upon request, select 'No' for the first question and explain your exceptional situation in the text box.<br><br>Do the authors confirm that all data underlying the findings described in their manuscript are fully available without restriction?	
**Describe where the data may be found in full sentences. If you are copying our sample text, replace any instances of XXX with the appropriate details.**<br><br>• If the data are **held or will be held in a public repository**, include URLs, accession numbers or DOIs. If this information will only be available after acceptance, indicate this by ticking the box below. For example: *All XXX files are available from the XXX database (accession number(s) XXX, XXX.).*<br>• If the data are all contained **within the manuscript and/or Supporting Information files**, enter the following: *All relevant data are within the manuscript and its Supporting Information files.*<br>• If neither of these applies but you are able to provide **details of access elsewhere**, with or without limitations, please do so. For example:<br><br>*Data cannot be shared publicly because of [XXX]. Data are available from the XXX Institutional Data Access / Ethics Committee (contact via XXX) for researchers who meet the criteria for access to confidential data.*<br><br>*The data underlying the results presented in the study are available from (include the name of the third party*	All relevant data are within the manuscript.

and contact information or URL).<br>• This text is appropriate if the data are owned by a third party and authors do not have permission to share the data.<br><br>* typeset	
Additional data availability information:	

Main Manuscript Click here to access/download;Manuscript;210813 Belakehal Manuscript.docx

Evaluation of the discriminatory power of spoligotyping and 19-locus mycobacterial interspersed repetitive unit-variable number of tandem repeat analysis (MIRU-VNTR) of *Mycobacterium bovis* strains isolated from cattle in Algeria

Faïza Belakehal[1*], Stefanie A. Barth[2,3*], Christian Menge[2], Hamdi T. Mossadak[1], Naïm Malek[4], Irmgard Moser[2,3]

[1] High National Veterinary School, Laboratory of Food Hygiene and Quality Insurance System, El-Alia, Oued Smar, Algeria.

[2] Friedrich-Loeffler-Institut/Federal Research Institute for Animal Health, Institute of Molecular Pathogenesis, Germany.

[3] National Reference Laboratory for Bovine Tuberculosis, at Friedrich-Loeffler-Institut/Federal Research Institute for Animal Health, Institute of Molecular Pathogenesis, Germany.

[4] Central Military Hospital, Department of Microbiology, Kouba, Algeria.

Authors

*Faiza Belakehal (faiza.belakehal@gmail.com), ORCID: 0000-0001-8087-4960

Laboratory of Food Hygiene and Quality Insurance System, High National Veterinary School, Rue Issad Abbes, 16111 El Alia, Oued Smar, Algiers, Algeria. Phone: +213 549161575

*Stefanie A. Barth (stefanie.barth@fli.de), ORCID: 0000-0003-4921-5056

Friedrich-Loeffler-Institut/Federal Research Institute for Animal Health, Institute of Molecular Pathogenesis, Naumburgerstr. 96a, 07743 Jena, Germany. Phone +49 3641 8042270

Christian Menge (christian.menge@fli.de)

Hamdi T. Mossadak (tm.hamdi@ensv.dz) ORCID: 0000-0002-9639-5208

Naïm Malek (malekn@outlook.fr)

Irmgard Moser (irmgard_moser@t-online.de) ORCID: 0000-0003-2796-6967

*Corresponding authors with affiliation and contact

Abstract

Bovine tuberculosis (bTB) caused by *Mycobacterium* (*M.*) *bovis* and *M. caprae* is a transmissible disease of livestock, notifiable to the World Organization for Animal Health (OIE). BTB particularly affects cattle and small ruminants and can be transmitted to humans thereby posing a significant threat to veterinary and public health worldwide. *M. bovis* is the principal cause of bTB in Algeria. In order to better understand the route of spreading and elaborate an eradication program, isolation and characterization of mycobacteria from Algerian cattle has been performed. Sixty strains belonging to the *M. tuberculosis* complex have been analyzed by spoligotyping, thereof 42 by 19-locus-MIRU-VNTR-typing. Spoligotyping revealed 16 distinguishable patterns (Hunter-Gaston discriminatory index [HGDI] of 0.8294), with types SB0120 (n = 20) and SB0121 (n = 13) being the most frequent patterns, representing 55% of the strains. Analyses based on 19-locus-MIRU-VNTR yielded 32 different profiles, five clusters and one orphan pattern, showing higher discriminatory power (HGDI = 0.9779) than spoligotyping. Seven VNTR-loci [VNTR 577 (alias ETR C), 2163b (QU11b), 2165 (ETR A), 2461 (ETR B), 3007 (MIRU 27), 2163a (QUB11a) and 3232 (QUB 3232)] were the most discriminative loci (HGDI > 0.50). In conclusion, 19-locus-MIRU-VNTR yielded more information than spoligotyping concerning molecular differentiation of strains and better supports the elucidation of transmission routes of *M. bovis* between Algerian cattle herds.

Keywords: Algeria, *Mycobacterium bovis, Mycobacterium caprae*, molecular characterization, spoligotyping, MIRU-VNTR-typing, cattle

Short title: *Mycobacterium bovis* from cattle in Algeria

Introduction

Bovine tuberculosis (bTB) represents a veterinary and public health threat of global relevance. Despite the importance of the disease, there is no official data concerning the true prevalence of bTB in many low and middle-income countries. This also holds for Algeria, despite the fact that bTB is a notifiable animal disease [1]. Although multiple disease control plans are implemented by the Algerian official's health services, there are still many obstacles to control and eradicate bTB. Skin test and meat inspection at slaughter represent the main methods in bTB surveillance. Application of culture and molecular diagnostic methods is extremely limited in low-income countries due to high costs and time requirements [2]. Several strategies assessing cost effectiveness and socio-economic factors have been proposed to fight bTB [3]. In Algeria, we suspect that most bTB-infected herds are geographically clustered in the north-east of the country because of the high concentration of cattle compared to other areas. It is assumed that transmission of bTB in Algeria is mainly attributed to cattle movement, although a study conducted in France by Palisson [4] disclosed the key role played by the spatial neighborhood of infected herds. Genotyping tools are crucial for unveiling pathogen transmission routes in bTB and tracing back sources of infections. The main goal of the present study was molecular typing of the Algerian *M. bovis* population in order to improve our understanding of disease epidemiology and bTB spread in Algeria. Spoligotyping [5, 6] and mycobacterial interspersed repetitive units-variable number of tandem repeats (MIRU-VNTR) analyses were used [7], since the combination of these two genetic markers is known to be a powerful tool to study the molecular epidemiology in a *M. bovis* population [8]. The DR region (direct repeat) analyzed by spoligotyping is a unique region (locus) which contains well-conserved 36 bp direct repeats separated by variable sequences from 35 to 41 bp. These sequences vary from one strain to another by their length, their sequence and their number. The differentiation of the strains is based on the variability of the number of DRs and on the presence

or absence of the particular spacers [5]. Because of its simplicity, its binary result format and its high reproducibility, spoligotyping is widely used for the molecular epidemiology of members of the *M. tuberculosis* complex (MTC) [9]. However, this method is less resolving than either VNTR typing or restriction enzyme analysis, limiting its usefulness in forensic investigations [10]. The VNTR loci containing a variable number of tandem repeats are amplified by PCR using specific primers and the size of the product is determined by gel electrophoresis. The MIRU-VNTR technique is faster than restriction fragment length polymorphism (RFLP) analysis, easy to interpret, more reproducible between laboratories [11] and gives a higher degree of discrimination than spoligotyping [12].

Materials and Methods

Bacterial strains and molecular identification

A total of 3,848 cattle were inspected for bTB at four slaughterhouses in the north part of Algeria between January and May 2017. The origin of the animals could not be determined due to the lack of an efficient livestock identification system in Algeria. The most important target organs for tuberculosis lesions, i.e. the respiratory tract (lung tissue and lymph nodes), the thoracic cavity, retropharyngeal lymph nodes, liver and kidney, were inspected at slaughter. Tissue samples were taken during inspection of carcasses for the presence of visible lesions suspected of tuberculosis. The cut surfaces of lymph node are examined carefully for the presence of abscess, caseous mass and tubercles. Out of 3,848 animals screened, 184 carcasses presented with lesions suspicious of bTB and samples from single organs of 105 animals were submitted to microbiological examination. Organs predominantly affected were respiratory tract and lymph nodes. The tissue samples were decontaminated according to the modified

Petroff's method [13, 14]. Briefly, an amount of 5 ml NaOH solution (4%) was added to about 10 g of tissue, minced by a mortar in 4 ml of sterile distilled water, and incubated for 15 min. The suspension was diluted by 10 ml of sterile distilled water and then centrifuged at 3000 x g for 20 min. The pellet was used as an inoculum to solid Löwenstein-Jensen medium. Mycobacteria were cultured at Pasteur Institute of Algiers (IPA), Reference Laboratory for Tuberculosis and Mycobacteria for six to eight weeks according to standard protocols.

The molecular characterization of isolates was performed at the National Reference Laboratory for Bovine Tuberculosis at the Friedrich-Loeffler-Institut, Jena, Germany. Using bacterial DNA extracted by the thermal lysis method described by Berg et al. [15], strains were identified as members of the MTC using the RD9 PCR. The PCR was performed using oligonucleotide primers for detection of the RD9 deletion. A PCR reaction mix consisted of 0.5 µl of each primer (100 µM), 0.2 µl of Taq polymerase (Taq PCR Core Kit, Qiagen, Germany), 2 µl 10x PCR buffer, 4 µl of Q solution, 0.4 µl of 0.25 mM $MgCl_2$, 0.4 µl dNTPs (10 mM each) and 1 µl of purified DNA in a final volume of 20 µl. PCR amplification program included one denaturation step (15 min, 96°C) followed by 35 cycles of 1 min at 95°C, 1 min at 60°C and 1 min at 72°C. PCR amplification products were separated in 1.5 % agarose gels after electrophoresis at 110 V for 1 h and visualized using ethidium bromide under UV light. As positive control served the strains *M. bovis* BCG (DSMZ 43990, ATCC 27289) and *M. tuberculosis* H37Rv.

Molecular typing

Spoligotyping was performed using the DNA microarray format of the ArrayStrip platform (Alere Technologies GmbH [now Abbott], Jena, Germany) according to the manufacturer's instructions as described by Ruettger et al. [6]. Briefly, after DNA extraction with the thermal lysis method, DR regions were amplified using 5'-biotinylated primers DRa/DRb [5], and then

allowed to hybridize on the microarray (hybridization at 60°C, wash steps at 55°C). Finally, arrays were scanned and analyzed by the ArrayMate™. The Alere Technologies software measures the signal intensity of each probe and compares the profiles automatically to those available at the *M. bovis* Spoligotype Database (www.mbovis.org [16]) and the SITVIT1 database (www.pasteur-guadeloupe.fr:8081/SITVIT_ONLINE/index.jsp [17]).

MIRU-VNTR typing of the *M. bovis* strains was carried out by in-house technique [18], according to Supply et al. [7] with slight modifications, as for VNTR locus 2461-PCR the primers described by Frothingham et al. [19] and for VNTR locus 2163a-PCR the primers of Skuce et al. [20] were used. DNA was purified using the High Pure PCR Template Preparation Kit, Roche Life Science (USA). The PCRs were performed using 0.8 µl of each primer (20 µM; MWG Eurofins, Germany), 0.1 µl of Taq polymerase (HotStarTaq DNA Polymerase, Qiagen, Germany), 2 µl 10x PCR buffer, 4 µl 5x Q solution, 0.4 µl 0.25 mM $MgCl_2$, 0.8 µl dNTPs (10 mM each) and 1 µl of purified DNA in a final volume of 20 µl. PCR amplification program included one denaturation step (15 min, 95°C) followed by 35 cycles of 1 min at 94°C, 1 min at 59°C and 120 s at 72°C. The PCR for VNTR locus 2163a differed with a 25 µl reaction mix (0.5 µl of each primer, 0.1 µl of Hotstar Taq polymerase, 2.5 µl 10x PCR buffer, 1.25 µl 5x Q solution, 0.5 µl dNTPs and 2 µl of purified DNA) and an annealing temperature of 55°C during amplification [20]. PCR amplification products were separated in 1.5 % agarose gels after electrophoresis at 100 V for 2 h 15 min and visualized using ethidium bromide under UV light.

Allelic diversity estimations were calculated using the Hunter-Gaston discriminatory index (HGDI) [21]. The HGDI was used to determine the allelic diversity within each MIRU-VNTR locus and the genotypic diversities (discriminatory power) of the spoligotyping assays, the 19-locus MIRU-VNTR and the combination of both methodologies, using the website application

http://insilico.ehu.es/. The sample profiles were analyzed by BioNumerics (version 7.6.2, Applied Maths, The Netherlands) to assess the overall genetic similarity of the strains.

Results and Discussion

Bacterial strains and molecular identification

Of a total of 105 samples submitted to microbiological examination, 60 samples showed bacterial growth on Löwenstein-Jensen medium. With a first screening by analysis for acid-fast bacteria, all 60 samples yielded a positive signal and were subsequently subjected to DNA extraction. Subsequent RD9 PCR showed that all 60 strains belonged to *M. tuberculosis* complex (MTC) whereas non-tuberculous mycobacteria were not isolated.

Spoligotyping

Of the 60 MTC positive strains, 59 were identified as *M. bovis* by spoligotyping, one as *M. caprae*. Among the 60 strains, 16 different spoligotypes were identified (Table 1). Four new spoligotypes have been found in the present study and were subsequently submitted to the *M. bovis* spoligotype database (www.mbovis.org). Those new spoligotypes received the ID numbers SB2520 (n = 1), SB2521 (n = 5), SB2522 (n = 1), and SB2523 (n = 1). Two of the four new spoligotypes, SB2520 and SB2521, have been described recently in Algerian cattle and human samples respectively [22].

Table 1. Spoligotype patterns of 59 *M. bovis* and one *M. caprae* strains and their relative frequencies.

SB No.	Spoligotype		No. of strains	Frequency [%]	SIT	Family
	binary code	octal code				
SB0120	1101111101111110111111111111111111111100000	676773777777600	20	33.3	482	BOV_1
SB0121	1101111101111110111101111111111111111100000	676773677777600	13	21.7	481	BOV_1
SB0134	1100011101111110111111111111111111111100000	616773777777600	7	11.7	665	BOV_1
SB2521	1100011101111110111011111111111111111100000	616773577777600	5	8.3	n.k.	n.k.
SB0339	1101111101111110111101111000011111111100000	676773674177600	2	3.3	696	BOV_1
SB0828	1101111101111110111111111111111110111100000	676773777773600	2	3.3	1047	BOV_1
SB1542	1100011100011110111111111111111111111100000	616173777777600	2	3.3	n.k.	BOV
SB0119	1101111101111100111101111111111111111100000	676763677777600	1	1.7	695	BOV
SB0818	1101111101111110111111111111111101111100000	676773777767600	1	1.7	1044	BOV_1
SB0822	1100111101111110111111111111111111111100000	636773777777600	1	1.7	997	BOV_1
SB0848	1101111101110110111101111111111111111100000	676733677777600	1	1.7	1010	BOV_1
SB1452	1101111000111100111101111111111111111100000	674363677777600	1	1.7	1595	BOV
SB2520	1100011101111110111111111110111111111100000	616773777377600	1	1.7	n.k.	n.k.
SB2522	1101111101111110111110011111111011111100000	676773717757600	1	1.7	n.k.	n.k.
SB2523	1101111101111110100111111111111111111100000	676772377777600	1	1.7	n.k.	n.k.
SB0835	0100000000000000111111111110111011111100000	200003777357600	1	1.7	978	BOV_4 Caprae
	Total		60	100		

n.k.: not known; **SIT:** spoligo-international type according to the SITVIT1 nomenclature; **BOV:** Bovine.

The binary code is represented by 43 digits. The number 0 indicate the absence of signal and the number 1 indicate the presence of signal.

The predominant spoligotypes found were SB0120 (n = 20) and SB0121 (n = 13), both belonging to the family BOV_1 and differing in only one spacer (no. 21), followed by SB0134 (n = 7), SB2521 (n = 5), and SB0828 (n = 2). The currently most prevalent spoligotypes SB0120, SB0121, and SB0134 were already detected by a study conducted 10 years earlier in Algeria [23]. Of note, the two new types SB2522 and SB2523 as well as three additional spoligotypes, namely SB0119 (n = 1), SB0848 (n = 1), and SB1542 (n = 2) have not been reported in Algeria before.

Presence of spoligotypes SB0121, SB0120, and SB0134 was also reported for the neighboring countries Morocco and Tunisia as well as France [24-26]. Interestingly, the second most frequently found spoligotype in Morocco, type SB0265, was not detected in our study in Algerian cattle although in silico analysis of whole genome sequence (WGS) data from four Algerian human *M. bovis* isolates [27] could assign two of the strains to SB0265 implying that the spoligotype is present in Algeria, too. The BCG cluster associated spoligotypes SB0120 and SB0121 are circulating nearly worldwide in Europe, Africa, and America [28, 29]. Spoligotype SB0134 is also frequent in many African countries (e.g., Tunisia, Morocco, Mali and Ethiopia) [30-33]. The new type SB2520 identified in our study has been recently found in Ethiopia [33]. The spoligotypes SB0339 and SB0119 are common on the Iberian peninsula, thereof SB0339 frequently reported for Spain and SB0119 in Portugal, and both were also reported for Morocco [24, 34, 35]. The spoligotype SB0818 has been reported in Italy [36] and France [26, 37], SB0848 in the neighboring country Tunisia [31] and in Portugal [38]. According to the database www.mbovis.org, spoligotype SB1542 was previously detected in Italy and *M. caprae* SB0835 in France. *M. caprae* has been identified mainly in continental Europe in the alpine region [39] and Spain [40]. In the study presented here only one *M. caprae* isolate with the spoligotype SB0835 was detected. Only few data exist on the detection of *M. caprae* in Africa. In Algeria

two isolates of spoligotypes SB1451 and SB0835 were described before [22, 23], pointing to a rare occurrence of *M. caprae* in this country.

Nowadays, the composition of the spoligotypes in North Africa (Morocco, Algeria, Tunisia) is dominated by spoligotypes SB120, SB121 and SB0134 possibly having evolved from common ancestral strains introduced since decades from Europe [23-25]. Indeed, this can be explained by recent imports, given the incomplete eradication of bTB in Europe. France represents the country from which the majority of cattle in Algeria were imported. According to WAHIS interface, France is declared "officially tuberculosis free" (OTF) by the EU but bTB still exists and single outbreaks are repeatedly reported for cattle herds and wildlife in some areas of the country. Thereby, SB0120, SB121 and SB0134 are the major spoligotypes distributed in animals in France, too [26]. Moreover, a study conducted in Tunisia by Siala and colleagues analysed human *M. bovis* isolates and found 13 diffferent spoligotypes, thereof, SB0120 and SB0121 as the dominating types [41].

MIRU-VNTR typing

Out of 60 strains of this study, VNTR profiles could be generated for 42 *M. bovis* strains. Missing success in the remaining cases might have been due to low DNA quality or quantity. A panel of 19 loci was chosen for conducting the MIRU-VNTR (Table 2).

Table 2. Allele diversity of the 19-locus MIRU-VNTR in 42 Algerian *M. bovis* strains.

**Locus**		**Number of strains with the respective number of copies**													**Allele diversity (HGDI)**
**VNTR**	**Alias**	**1**	**2**	**3**	**4**	**5**	**6**	**7**	**8**	**9**	**10**	**11**	**12-15**	**16**	
2165	ETR-A	0	0	**1**	**7**	**11**	**15**	**8**	0	0	0	0	0	0	0.7573
3232	QUB3232	0	0	**2**	**1**	0	**17**	**13**	**8**	**1**	0	0	0	0	0.7178
2163a	QUB11a	**2**	0	0	0	**1**	0	0	0	**15**	**17**	**5**	0	**1**	0.6927
2163b	QUB11b	0	**16**	**8**	**16**	**1**	0	**1**	0	0	0	0	0	0	0.6887
2461	ETR-B	0	0	**1**	**20**	**19**	**2**	0	0	0	0	0	0	0	0.5796
3007	MIRU27	**3**	**17**	**22**	0	0	0	0	0	0	0	0	0	0	0.5703
577	ETR-C	**1**	**1**	**13**	**2**	**25**	0	0	0	0	0	0	0	0	0.5598
4052	QUB26	**3**	**1**	**2**	**5**	**30**	**1**	0	0	0	0	0	0	0	0.4785
3192	MIRU31/ETR-E	0	**3**	**33**	**6**	0	0	0	0	0	0	0	0	0	0.3659
580	MIRU04/ETR-D	**2**	0	**38**	**1**	0	0	0	0	**1**	0	0	0	0	0.1823
2687	MIRU24	**2**	**40**	0	0	0	0	0	0	0	0	0	0	0	0.0929
154	MIRU02	**1**	**41**	0	0	0	0	0	0	0	0	0	0	0	0.0476
802	MIRU40	**1**	**41**	0	0	0	0	0	0	0	0	0	0	0	0.0476
2059	MIRU20	**1**	**41**	0	0	0	0	0	0	0	0	0	0	0	0.0476
2996	MIRU26	0	0	0	**1**	**41**	0	0	0	0	0	0	0	0	0.0476
4348	MIRU39	0	**41**	**1**	0	0	0	0	0	0	0	0	0	0	0.0476
960	MIRU10	0	**42**	0	0	0	0	0	0	0	0	0	0	0	0
1644	MIRU16	0	0	**42**	0	0	0	0	0	0	0	0	0	0	0
2531	MIRU23	0	0	0	**42**	0	0	0	0	0	0	0	0	0	0

ETR: Exact Tandem Repeat; **MIRU:** Mycobacterial Interspersed Repetitive Unit; **QUB:** Queen's University Belfast.

Evaluated loci were selected according to the 12-locus panel proposed in the MIRU-VNTR plus web site (www.miru-vntrplus.org) in combination with the panel proposed by the European Reference Center for Bovine Tuberculosis (EU-RL bTB, VISAVET Health Surveillance Centre, Universidad Complutense de Madrid) for the molecular typing of *M. bovis* in Europe. Among them, the ETR loci (VNTR580, 2165, 2461, and 3192) had been evaluated by Sahraoui et al. [42] for Algerian strains previously. Additionally, the QUB loci (VNTR2163a, 2163b,

3232, and 4052) and VNTR2996 were included as these are regarded highly discriminative for *M. bovis* strains.

Overall, seven loci (VNTR577, 2163a, 2163b, 2165, 2461, 3007, and 3232) presented a high discriminatory power (HGDI > 0.50, Table 2). Our findings corroborate therefore earlier data obtained in Tunisia [25] which showed a high resolution of VNTR2163a, 2163b, 2461, 2165, and 3232, while the four loci VNTR577, 2165, 2461, and 3007, showed a high discriminatory power for Algerian strains also in previous data sets [42]. Furthermore, this is consistent with data reported for *M. bovis* strains from Mexico and Germany [43, 44]. Rodriguez-Campos et al. [45] found only a very low allele diversity for locus VNTR2163b (HGDI = 0.08), but analyzed only SB0121 strains. The two loci VNTR3192 and 4052 showed moderate allelic diversity (HGDI = 0.36 and HGDI = 0.47, respectively) and locus VNTR580 a low allelic diversity (HGDI = 0.18). Very low allele diversity (HGDI <0.15) was observed for six loci (VNTR154, 802, 2059, 2687, 2996, and 4348). By contrast, Yang et al. [46], found a high allele diversity in the loci VNTR580 (HGDI = 0.607) and 802 (HGDI = 0.495) but no resolution for loci VNTR2059, 2687 and 2996. This difference may be related especially to the geographic region from which the strains have been isolated, as we analyzed exclusively bovine strains from Algeria whereas Yang et al. typed strains from Sika deer in Northern China [46]. Finally, there is a difference in the resolution of loci between MTC species. For example, loci VNTR960 and 2531 are reported very discriminatory for *M. tuberculosis* [47], but performed less well for *M. bovis* in the present study. VNTR2996 demonstrates a very low allele diversity in the present study (HGDI = 0.047). A possible explanation could be, that the number of strains analyzed in the present study was smaller and therefore less variance found. No allele diversity (HGDI = 0) was observed with the loci VNTR960, 1644, and 2531 which suggests that these loci are not suitable for typing Algerian *M. bovis* strains. Overall, analysis of 19-locus MIRU-VNTR in 42

strains revealed 32 different profiles among which 27 were found only in a single strain (Table 3).

Table 3. Comparison of the discriminatory power of spoligotyping, 19-locus MIRU-VNTR typing and the combination of both methods.

Variability	Spoligotyping	MIRU-VNTR	Spoligotyping and MIRU-VNTR
**No. of strains included**	60	42	42
**Total number of profiles (n)**	16	32	33
**Number of individual profiles (n)**	9	27	28
**Discriminatory index (HGDI)**	0.8294	0.9779	0.9826

The combination of MIRU-VNTR allele diversity and spoligotyping pattern demonstrated a large genotypic variety resulting in 33 profiles (Fig 1). Thereby spoligotypes were typed in sub-profiles, e.g. SB0120 in 13 sub-profiles, SB0121 in five, and SB0134 in five, reflecting the heterogeneity of strains causing bTB in Algeria.

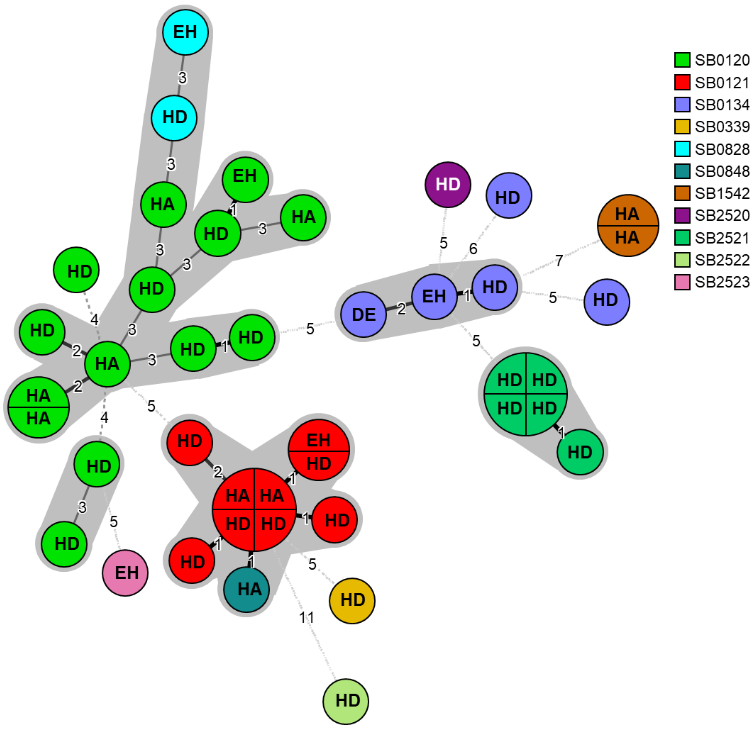

Fig 1. Minimum-spanning-tree based on the combination of spoligotyping and MIRU-VNTR results of 42 *M. bovis* strains isolated from slaughtered cattle in Algeria.
Each node represents a unique spoligotype-VNTR sub-profile. If more than one strain exhibited the identical profile the node is separated. Nodes closer than three differences are put into one cluster (gray background). The number of differing alleles (repeat number of MIRU-VNTR and absence/presence of spacer for spoligotyping) between two nodes is indicated by the respective number. The letter inside the nodes represent the slaughterhouse the strains came from (**HD**: Hussein Dey, **HA**: Hadjout, **EH**: El Harrach, **DE**: Dellys) (BioNumerics, version 7.6.2).

Scientific literature describes several routes of transmission of animal tuberculosis [48, 49]. The emergence of tuberculosis in wildlife [50] may constitute a continuous source for reinfection of cattle and could be at the origin of the persistence of bTB worldwide. This particularly holds for wild boar, which represent a new threat for livestock animals and agriculture in Algeria. Hence, the pathway of transmission of this pathogen in Algeria including wildlife needs to be studied. El Mrini et al. [51] described cases of tuberculosis due to *M. bovis* in Eurasian wild boar in Morocco. Without neglecting the role of re-infections within single cattle herds,

mycobacteria may survive decades unrecognized in several biological niches due to the chronic subclinical course of infection, leading to persisting bTB in Algerian livestock. On the other hand, all our *M. bovis* strains were recovered from lungs and associated lymph nodes, suggesting a primarily airborne transmission of bTB to the cattle under study here and, consequently, an intimate contact between the respective animals. Indeed, combination of MIRU-VNTR allele diversity and spoligotyping pattern allowed us to unveil some possible epidemiological links. Constructing a minimum-spanning-tree revealed five clusters (spoligotype and MIRU-VNTR profile differing in 3 allels in maximum) with the largest one comprising of 14 strains. Two sub-profiles (one in the SB0121 cluster and the other in the SB2521 cluster) contained four strains, that were identical in their spoligotype and MIRU-VNTR profiles, two additional sub-profiles were represented by two strains each. The four strains of the SB2521 sub-profile were all isolated at the slaughterhouse Hussein Dey. It is tempting to assume that cross-infection occurred between animals within one herd or the same area. In contrast, both sub-profiles with four and two strains, respectively, within the spoligotype SB0121 contained strains probably from different areas in Algeria as the strains were isolated from cattle slaughtered in different abattoirs. This includes the slaughterhouses of Hussein Dey and Hadjout which are 88 km distant from each other (source of the strains belonging to the four strain sub-profile), as well as slaughterhouses in El Harrach and Hussein Dey which are more proximate to each other. As we consider it unlikely that one owner had delivered his cattle to different slaughterhouses, the occurrence of identical spoligotype/MIRU-VNTR profiles in different geographical areas might be explained by the trade of animals between herds in those areas. In two cluster, two different spoligotypes (SB0848 and SB0121 as well as SB0828 and SB0120) are combined. Both pairs differ from each other only in one spacer (no. 13 is missing in SB0848 compared to SB0121 as well as no. 34 is missing in SB0282 compared to SB0120) it might be possible that spoligotypes SB0828 and SB0848 may have

originated from spoligotype SB0120 and SB0121, respectively, by undergoing a genetic mutation on spacer 13 respectively 34. The singular SB0848 strain and four SB0121 strains possess identical MIRU-VNTR profiles, supporting the close relatedness of both spoligotypes.

Beyond disclosing recent transmission events and microevolution of *M. bovis* strains, genotyping of bacteria also may allow for a reconstruction of phylogeography and evolutionary history of these pathogens [52]. Spoligotyping in combination with other genomic markers, such as deletions [52, 53] and single-nucleotide polymorphisms (SNPs) have been used to construct a phylogeography for *M. bovis* strains. The clonal complexes named "African 1" (Af1, characterized by missing of spacer 30) and "African 2" (Af2, missing spacers 3-7) were described as geographically localized in Central-West and East Africa, respectively. The cluster "European 1" (Eu1, missing spacer 11) is found mainly on the British Islands, in New Zealand, Australia, South Africa, and Korea and "European 2" (Eu2, missing spacer 21) on the Iberian peninsula and Western Europe [52-54]. Additional to those four 'classical' phylogenetic lineages recently eight new clonal lineages were defined on the basis of WGS data of more than 3,300 *M. bovis* isolates and spoligotypes where affiliated to certain phylogenetic lines [52]. Six out of the 16 spoligotypes detected in our study are also mentioned in this recent publication. The discriminatory capacity of spoligotyping is limited since diversity is measured at a single locus prone to convergent evolution and phylogenetic distances cannot be reliably inferred [55]. Despite this limitation our findings might point towards the phylogenetic positions of strains and indicate the multiplicity of clonal lines circulating in Algeria. The signature profile of the *M. bovis* BCG vaccine strain is the absence of spacers 3, 9, 16, and 39 to 43 [29]. The BCG cluster is divided into two groups: the BCG-like group, represented by SB0121, and the ancestor BCG-like SB0120. In the present study, all *M. bovis* strains lacked spacers 3, 9, 16, and 39 to 43 and were therefore grouped to the BCG cluster (Table 1). On the African continent, the ancestral BCG-like cluster is predominantly found in Algeria, Zambia [56], and Mozambique

[57]. The cluster Af1, Af2, and Eu1 have not been found in cattle outside of the previous mentioned regions [24]. Further studies, implicating WGS technology, will be required to determine if or to what extend the current bTB epizootic in Algeria originates from the long-term evolution during which animal-adapted MTC, i.e. *M. caprae* and *M. bovis*, might have originally come out of Africa [58] or on a more recent (re)introduction of *M. bovis* from Europe as discussed above.

Conclusion

Until now, tuberculosis still represents a serious burden in Algeria for both, humans and animals. The BCG cluster derived spoligotypes (SB0120, SB0121) are the most frequent types circulating in Algeria and also worldwide. The analysis of four MIRU-VNTR loci [ETR A, VNTR2163b (QUB11b), 2163a (QUB11a), and 3232 (QUB3232)] would be sufficient to characterize Algerian *M. bovis* strains deeply. Nevertheless, the combination of MIRU-VNTR and spoligotyping is highly advisable. The association of those two techniques demonstrates the heterogenic population of Algerian *M. bovis* strains, indicating that different strains are responsible for bTB in Algeria. However, the lack of information concerning the origin of cattle (herd, country) currently impairs the unveiling of transmission routes of bTB in Algeria and must be implemented as part of the control program for the eradication of bovine tuberculosis in Algeria.

Acknowledgments

The author’s address special thanks to the technicians Uta Brommer and Josefine Bach at FLI, Jena, Germany, for their excellent support to perform the molecular methods. We are grateful

to Prof. Dr. Dr. h.c. Thomas C. Mettenleiter for providing the opportunity to perform the practical part of this work at the FLI, Jena, Germany.

References

1. Journal Officiel de la Republique Algerienne (JORA) N°16. Decret exécutif n°06-118 du 12 Safar 1427 correspondant au 12 Mars 2006 complétant le décret exécutif n°88-252 du 31 Décembre 1988 fixant les conditions d’exercice à titre privé des activités de médecine vétérinaire et de chirurgie des animaux. 2006-03-15.
2. Asebe G. Molecular Characterization of Mycobacterium bovis and its Significance: Role for Control of Zoonotic Tuberculosis in Africa. Journal of Medical Diagnostic Methods. 2017;06(03):250. doi: 10.4172/2168-9784.1000250.
3. Riviere J, Le Strat Y, Hendrikx P, Dufour B. Cost-effectiveness evaluation of bovine tuberculosis surveillance in wildlife in France (Sylvatub system) using scenario trees. PLoS One. 2017;12(8):e0183126. Epub 2017/08/12. doi: 10.1371/journal.pone.0183126. pmid: 28800642.
4. Palisson A, Courcoul A, Durand B. Role of Cattle Movements in Bovine Tuberculosis Spread in France between 2005 and 2014. PLoS One. 2016;11(3):e0152578. Epub 2016/03/29. doi: 10.1371/journal.pone.0152578. pmid: 27019291.
5. Kamerbeek J, Schouls L, Kolk A, van Agterveld M, van Soolingen D, Kuijper S, et al. Simultaneous detection and strain differentiation of Mycobacterium tuberculosis for diagnosis and epidemiology. J Clin Microbiol. 1997;35(4):907-14. pmid: 9157152.
6. Ruettger A, Nieter J, Skrypnyk A, Engelmann I, Ziegler A, Moser I, et al. Rapid spoligotyping of Mycobacterium tuberculosis complex bacteria by use of a microarray system with automatic data processing and assignment. J Clin Microbiol. 2012;50(7):2492-5. doi: 10.1128/JCM.00442-12. pmid: 22553239.
7. Supply P, Allix C, Lesjean S, Cardoso-Oelemann M, Rusch-Gerdes S, Willery E, et al. Proposal for standardization of optimized mycobacterial interspersed repetitive unit-variable-number tandem repeat typing of *Mycobacterium tuberculosis*. J Clin Microbiol. 2006;44(12):4498-510. doi: 10.1128/JCM.01392-06. pmid: 17005759.
8. Marianelli C, Amato B, Boniotti MB, Vitale M, Pruiti Ciarello F, Pacciarini ML, et al. Genotype diversity and distribution of Mycobacterium bovis from livestock in a small, high-risk area in northeastern Sicily, Italy. PLoS Negl Trop Dis. 2019;13(7):e0007546. Epub 2019/07/16. doi: 10.1371/journal.pntd.0007546. pmid: 31306431.
9. Couvin D, Rastogi N. La mise en place de bases de données des génotypes circulants du complexe Mycobacterium tuberculosis et des outils web permettant de mieux surveiller, comprendre et contrôler l'épidémie de la tuberculose dans le monde. 2014;2014(12):pp. 40-53.

10. Kao RR, Price-Carter M, Robbe-Austerman S. Use of genomics to track bovine tuberculosis transmission. Rev Sci Tech. 2016;35(1):241-58. Epub 2016/05/25. doi: 10.20506/rst.35.1.2430. pmid: 27217181.

11. Duignan A, Good M, More SJ. Quality control in the national bovine tuberculosis eradication programme in Ireland. Rev Sci Tech. 2012;31(3):845-60. Epub 2013/03/26. doi: 10.20506/rst.31.3.2166. pmid: 23520738.

12. Durr PA, Hewinson RG, Clifton-Hadley RS. Molecular epidemiology of bovine tuberculosis. I. Mycobacterium bovis genotyping. Rev Sci Tech. 2000;19(3):675-88. Epub 2000/01/11. doi: 10.20506/rst.19.3.1241. pmid: 11107611.

13. Kent PT, Kubica GP. Public Health Mycobacteriology: A Guide for the Level III Laboratory. Centers for Disease Control, Atlanta, GA, 1985.

14. Tripathi K, Tripathi P, Nema S, Shrivastava A, Dwiwedi K, Dhanvijay A. Modified Petroff's Method: an Excellent Simplified Decontamination Technique in Comparison with Petroff's Method. International Journal of Recent Trends in Science And Technology. 2014;10(3):461-4.

15. Berg S, Firdessa R, Habtamu M, Gadisa E, Mengistu A, Yamuah L, et al. The burden of mycobacterial disease in ethiopian cattle: implications for public health. PLoS One. 2009;4(4):e5068. doi: 10.1371/journal.pone.0005068. pmid: 19352493.

16. Smith NH, Upton P. Naming spoligotype patterns for the RD9-deleted lineage of the Mycobacterium tuberculosis complex; www.Mbovis.org. Infect Genet Evol. 2012;12(4):873-6. doi: 10.1016/j.meegid.2011.08.002. pmid: 21855653.

17. Demay C, Liens B, Burguiere T, Hill V, Couvin D, Millet J, et al. SITVITWEB--a publicly available international multimarker database for studying Mycobacterium tuberculosis genetic diversity and molecular epidemiology. Infect Genet Evol. 2012;12(4):755-66. Epub 2012/03/01. doi: 10.1016/j.meegid.2012.02.004. pmid: 22365971.

18. Kohl TA, Utpatel C, Niemann S, Moser I. Mycobacterium bovis Persistence in Two Different Captive Wild Animal Populations in Germany: a Longitudinal Molecular Epidemiological Study Revealing Pathogen Transmission by Whole-Genome Sequencing. J Clin Microbiol. 2018;56(9). Epub 2018/06/29. doi: 10.1128/JCM.00302-18. pmid: 29950330.

19. Frothingham R, Meeker-O'Connell WA. Genetic diversity in the Mycobacterium tuberculosis complex based on variable numbers of tandem DNA repeats. Microbiology. 1998;144 (Pt 5):1189-96. doi: 10.1099/00221287-144-5-1189. pmid: 9611793.

20. Skuce RA, McCorry TP, McCarroll JF, Roring SMM, Scott AN, Brittain D, et al. Discrimination of Mycobacterium tuberculosis complex bacteria using novel VNTR-PCR targets. Microbiology (Reading). 2002;148(Pt 2):519-28. Epub 2002/02/08. doi: 10.1099/00221287-148-2-519. pmid: 11832515.

21. Hunter PR, Gaston MA. Numerical index of the discriminatory ability of typing systems: an application of Simpson's index of diversity. J Clin Microbiol. 1988;26(11):2465-6. Epub 1988/11/01. doi: 10.1128/jcm.26.11.2465-2466.1988. pmid: 3069867.

22. Damene H, Tahir D, Diels M, Berber A, Sahraoui N, Rigouts L. Broad diversity of Mycobacterium tuberculosis complex strains isolated from humans and cattle in Northern Algeria suggests a zoonotic transmission cycle. PLoS Negl Trop Dis. 2020;14(11):e0008894. Epub 2020/12/01. doi: 10.1371/journal.pntd.0008894. pmid: 33253150.

23. Sahraoui N, Muller B, Guetarni D, Boulahbal F, Yala D, Ouzrout R, et al. Molecular characterization of Mycobacterium bovis strains isolated from cattle slaughtered at two abattoirs in Algeria. BMC Vet Res. 2009;5:4. Epub 2009/01/29. doi: 10.1186/1746-6148-5-4. pmid: 19173726.

24. Yahyaoui-Azami H, Aboukhassib H, Bouslikhane M, Berrada J, Rami S, Reinhard M, et al. Molecular characterization of bovine tuberculosis strains in two slaughterhouses in Morocco. Bmc Veterinary Research. 2017;13. doi: 10.1186/s12917-017-1165-6. pmid: WOS:000408325200001.

25. Djemal SE, Siala M, Smaoui S, Kammoun S, Marouane C, Bezos J, et al. Genetic diversity assessment of Tunisian Mycobacterium bovis population isolated from cattle. BMC Vet Res. 2017;13(1):393. Epub 2017/12/17. doi: 10.1186/s12917-017-1314-y. pmid: 29246228.

26. Hauer A, De Cruz K, Cochard T, Godreuil S, Karoui C, Henault S, et al. Genetic Evolution of Mycobacterium bovis Causing Tuberculosis in Livestock and Wildlife in France since 1978. Plos One. 2015;10(2). doi: 10.1371/journal.pone.0117103. pmid: WOS:000349444900101.

27. Tazerart F, Saad J, Niar A, Sahraoui N, Drancourt M. Mycobacterium bovis Pulmonary Tuberculosis, Algeria. Emerg Infect Dis. 2021;27(3):972-4. Epub 2021/02/25. doi: 10.3201/eid2703.191823. pmid: 33622469.

28. Ghavidel M, Mansury D, Nourian K, Ghazvini K. The most common spoligotype of Mycobacterium bovis isolated in the world and the recommended loci for VNTR typing; A systematic review. Microb Pathog. 2018;118:310-5. Epub 2018/03/27. doi: 10.1016/j.micpath.2018.03.036. pmid: 29578066.

29. Haddad N, Masselot M, Durand B. Molecular differentiation of Mycobacterium bovis isolates. Review of main techniques and applications. Res Vet Sci. 2004;76(1):1-18. Epub 2003/12/09. doi: 10.1016/s0034-5288(03)00078-x. pmid: 14659724.

30. Biffa D, Skjerve E, Oloya J, Bogale A, Abebe F, Dahle U, et al. Molecular characterization of Mycobacterium bovis isolates from Ethiopian cattle. BMC Vet Res. 2010;6:28. Epub 2010/05/29. doi: 10.1186/1746-6148-6-28. pmid: 20507576.

31. Lamine-Khemiri H, Martinez R, Garcia-Jimenez WL, Benitez-Medina JM, Cortes M, Hurtado I, et al. Genotypic characterization by spoligotyping and VNTR typing of Mycobacterium bovis and Mycobacterium caprae isolates from cattle of Tunisia. Trop Anim Health Prod. 2014;46(2):305-11. Epub 2013/10/26. doi: 10.1007/s11250-013-0488-y. pmid: 24158359.

32. Muller B, Steiner B, Bonfoh B, Fane A, Smith NH, Zinsstag J. Molecular characterisation of Mycobacterium bovis isolated from cattle slaughtered at the Bamako abattoir in Mali. BMC Vet Res. 2008;4:26. Epub 2008/07/19. doi: 10.1186/1746-6148-4-26. pmid: 18637160.

33. Mekonnen GA, Mihret A, Tamiru M, Hailu E, Olani A, Aliy A, et al. Genotype Diversity of Mycobacterium bovis and Pathology of Bovine Tuberculosis in Selected Emerging Dairy Regions of Ethiopia. Front Vet Sci. 2020;7:553940. Epub 2020/11/17. doi: 10.3389/fvets.2020.553940. pmid: 33195524.

34. de la Fuente J, Diez-Delgado I, Contreras M, Vicente J, Cabezas-Cruz A, Tobes R, et al. Comparative Genomics of Field Isolates of Mycobacterium bovis and M. caprae Provides

Evidence for Possible Correlates with Bacterial Viability and Virulence. PLoS Negl Trop Dis. 2015;9(11):e0004232. doi: 10.1371/journal.pntd.0004232. pmid: 26583774.

35. Matos F, Amado A, Botelho A. Molecular typing of Mycobacterium bovis isolated in the first outbreak of bovine tuberculosis in the Azores Islands: a case report. Vet Med (Praha). 2010;55(3):133-6. doi: 10.17221/137/2009-Vetmed. pmid: WOS:000276716000006.

36. Boniotti MB, Goria M, Loda D, Garrone A, Benedetto A, Mondo A, et al. Molecular typing of Mycobacterium bovis strains isolated in Italy from 2000 to 2006 and evaluation of variable-number tandem repeats for geographically optimized genotyping. J Clin Microbiol. 2009;47(3):636-44. Epub 2009/01/16. doi: 10.1128/JCM.01192-08. pmid: 19144792.

37. Boschiroli ML, Michelet L, Hauer A, de Cruz K, Courcoul A, Hénault S, et al. Tuberculose bovine en France : cartographie des souches de Mycobacterium bovis entre 2000-2013. Bulletin épidémiologique, santé animale et alimentation. 2015;70:2-8.

38. Matos F, Cunha MV, Canto A, Albuquerque T, Amado A, Botelho A. Snapshot of Mycobacterium bovis and Mycobacterium caprae infections in livestock in an area with a low incidence of bovine tuberculosis. J Clin Microbiol. 2010;48(11):4337-9. Epub 2010/09/17. doi: 10.1128/JCM.01762-10. pmid: 20844227.

39. Prodinger WM, Brandstatter A, Naumann L, Pacciarini M, Kubica T, Boschiroli ML, et al. Characterization of *Mycobacterium caprae* isolates from Europe by mycobacterial interspersed repetitive unit genotyping. J Clin Microbiol. 2005;43(10):4984-92. doi: 10.1128/JCM.43.10.4984-4992.2005. pmid: 16207952.

40. Rodriguez S, Bezos J, Romero B, de Juan L, Alvarez J, Castellanos E, et al. Mycobacterium caprae infection in livestock and wildlife, Spain. Emerg Infect Dis. 2011;17(3):532-5. doi: 10.3201/eid1703.100618. pmid: 21392452.

41. Siala M, Cassan C, Smaoui S, Kammoun S, Marouane C, Godreuil S, et al. A first insight into genetic diversity of Mycobacterium bovis isolated from extrapulmonary tuberculosis patients in South Tunisia assessed by spoligotyping and MIRU VNTR. PLoS Negl Trop Dis. 2019;13(9):e0007707. Epub 2019/09/19. doi: 10.1371/journal.pntd.0007707. pmid: 31532767.

42. Sahraoui N, Muller B, Djamel Y, Fadela B, Rachid O, Jakob Z, et al. Evaluation des VNTR (variable number of tandem repeats) des isolats de Mycobacterium bovis en Algerie. Ann Biol Clin (Paris). 2010;68(4):449-53. Epub 2010/07/24. doi: 10.1684/abc.2010.0459. pmid: 20650740.

43. Bolado-Martinez E, Benavides-Davila I, Candia-Plata Mdel C, Navarro-Navarro M, Aviles-Acosta M, Alvarez-Hernandez G. Proposal of a Screening MIRU-VNTR Panel for the Preliminary Genotyping of Mycobacterium bovis in Mexico. Biomed Res Int. 2015;2015:416479. Epub 2015/05/07. doi: 10.1155/2015/416479. pmid: 25945333.

44. Kohl TA, Kranzer K, Andres S, Wirth T, Niemann S, Moser I. Population structure of Mycobacterium bovis in Germany: A long-term study using Whole Genome Sequencing combined with conventional molecular typing methods. J Clin Microbiol. 2020. Epub 2020/08/21. doi: 10.1128/JCM.01573-20. pmid: 32817084.

45. Rodriguez-Campos S, Navarro Y, Romero B, de Juan L, Bezos J, Mateos A, et al. Splitting of a prevalent Mycobacterium bovis spoligotype by variable-number tandem-repeat typing reveals high heterogeneity in an evolving clonal group. J Clin Microbiol. 2013;51(11):3658-65. Epub 2013/08/30. doi: 10.1128/JCM.01271-13. pmid: 23985914.

46. Yang L, Wang C, Wang H, Meng Q, Wang Q. Evaluation of MIRU-VNTR for typing of Mycobacterium bovis isolated from Sika deer in Northeast China. BMC Vet Res. 2015;11:93. Epub 2015/04/19. doi: 10.1186/s12917-015-0402-0. pmid: 25889075.

47. Bouklata N, Supply P, Jaouhari S, Charof R, Seghrouchni F, Sadki K, et al. Molecular Typing of Mycobacterium Tuberculosis Complex by 24-Locus Based MIRU-VNTR Typing in Conjunction with Spoligotyping to Assess Genetic Diversity of Strains Circulating in Morocco. PLoS One. 2015;10(8):e0135695. Epub 2015/08/19. doi: 10.1371/journal.pone.0135695. pmid: 26285026.

48. Cowie CE, Hutchings MR, Barasona JA, Gortázar C, Vicente J, White PCL. Interactions between four species in a complex wildlife: livestock disease community: implications for Mycobacterium bovis maintenance and transmission. Eur J Wildlife Res. 2015;62(1):51-64. doi: 10.1007/s10344-015-0973-x.

49. Katale BZ, Mbugi EV, Siame KK, Keyyu JD, Kendall S, Kazwala RR, et al. Isolation and Potential for Transmission of Mycobacterium bovis at Human-livestock-wildlife Interface of the Serengeti Ecosystem, Northern Tanzania. Transbound Emerg Dis. 2017;64(3):815-25. Epub 2015/11/14. doi: 10.1111/tbed.12445. pmid: 26563417.

50. Aranaz A, De Juan L, Montero N, Sanchez C, Galka M, Delso C, et al. Bovine tuberculosis (Mycobacterium bovis) in wildlife in Spain. J Clin Microbiol. 2004;42(6):2602-8. Epub 2004/06/09. doi: 10.1128/JCM.42.6.2602-2608.2004. pmid: 15184440.

51. El Mrini M, Kichou F, Kadiri A, Berrada J, Bouslikhane M, Cordonnier N, et al. Animal tuberculosis due to Mycobacterium bovis in Eurasian wild boar from Morocco. Eur J Wildlife Res. 2016;62(4):479-82. doi: 10.1007/s10344-016-1022-0.

52. Loiseau C, Menardo F, Aseffa A, Hailu E, Gumi B, Ameni G, et al. An African origin for Mycobacterium bovis. Evol Med Public Health. 2020;2020(1):49-59. Epub 2020/03/27. doi: 10.1093/emph/eoaa005. pmid: 32211193.

53. Smith NH. The global distribution and phylogeography of Mycobacterium bovis clonal complexes. Infection Genetics and Evolution. 2012;12(4):857-65. doi: 10.1016/j.meegid.2011.09.007. pmid: WOS:000304219200035.

54. Rodriguez-Campos S, Smith NH, Boniotti MB, Aranaz A. Overview and phylogeny of Mycobacterium tuberculosis complex organisms: Implications for diagnostics and legislation of bovine tuberculosis. Res Vet Sci. 2014;97:S5-S19. doi: 10.1016/j.rvsc.2014.02.009. pmid: WOS:000345809400002.

55. Comas I, Homolka S, Niemann S, Gagneux S. Genotyping of genetically monomorphic bacteria: DNA sequencing in Mycobacterium tuberculosis highlights the limitations of current methodologies. PLoS One. 2009;4(11):e7815. Epub 2009/11/17. doi: 10.1371/journal.pone.0007815. pmid: 19915672.

56. Malama S, Johansen TB, Muma JB, Munyeme M, Mbulo G, Muwonge A, et al. Characterization of Mycobacterium bovis from Humans and Cattle in Namwala District, Zambia. Vet Med Int. 2014;2014:187842. Epub 2014/05/23. doi: 10.1155/2014/187842. pmid: 24847441.

57. Machado A, Rito T, Ghebremichael S, Muhate N, Maxhuza G, Macuamule C, et al. Genetic diversity and potential routes of transmission of Mycobacterium bovis in Mozambique. PLoS Negl Trop Dis. 2018;12(1):e0006147. Epub 2018/01/19. doi: 10.1371/journal.pntd.0006147. pmid: 29346413.

58. Brites D, Loiseau C, Menardo F, Borrell S, Boniotti MB, Warren R, et al. A New Phylogenetic Framework for the Animal-Adapted Mycobacterium tuberculosis Complex. Frontiers in microbiology. 2018;9:2820. Epub 2018/12/13. doi: 10.3389/fmicb.2018.02820. pmid: 30538680.
