# Supplementary material for: Evaluation of the discriminatory power of spoligotyping and 19-locus mycobacterial interspersed repetitive unit-variable number of tandem repeat analysis (MIRU-VNTR) of Mycobacterium bovis strains isolated from cattle in Algeria
Source: PLoS One. 2022 Jan 11;17(1):e0262390. doi: 10.1371/journal.pone.0262390 (PMC8751994; doi:10.1371/journal.pone.0262390)
Supplement: Attachment — Submitted filename: 211221 Rebuttal letter.docx [file pone.0262390.s003.docx]

Dr. Stefanie Barth

Friedrich-Loeffler Institut/Federal Research Institute for Animal Health,
Institute of Molecular Pathogenesis,
National Reference Laboratory for Bovine Tuberculosis,
Naumburger Strasse 96a,
07743 Jena, Germany

stefanie.barth@fli.de

Jena, 21^st^ of December 2021

**Rebuttal letter**

Dear Sirs,

on behalf of my colleagues, we wish to submit the revised version of the manuscript entiteled „Evaluation of the discriminatory power of spoligotyping and 19-locus mycobacterial interspersed repetitive unit-variable number of tandem repeat analysis (MIRU-VNTR) of *Mycobacterium bovis* strains isolated from cattle in Algeria” (PONE-D-21-26506).

We would like to thank the editior and the reviewer for their constructive comments and would like to answer point by point. Additionally, we highlighted all changes in the manuscript.

## Journal requirements:

1. Please ensure that your manuscript meets PLOS ONE's style requirements, including those for file naming. The PLOS ONE style templates can be found at <https://journals.plos.org/plosone/s/file?id=wjVg/PLOSOne_formatting_sample_main_body.pdf> and <https://journals.plos.org/plosone/s/file?id=ba62/PLOSOne_formatting_sample_title_authors_affiliations.pdf>.

We updated the title page and the style of the headings in the manuscript in order to meet all requirements.

2. In your Methods section, please provide the name of the slaughterhouse where the animals were sacrificed.

The names of the corresponding slaughterhouses have been provided in the method section of the paper as well as in the supporting information S1 Table.

3. We note that you are reporting an analysis of a microarray, next-generation sequencing, or deep sequencing data set. PLOS requires that authors comply with field-specific standards for preparation, recording, and deposition of data in repositories appropriate to their field. Please upload these data to a stable, public repository (such as ArrayExpress, Gene Expression Omnibus (GEO), DNA Data Bank of Japan (DDBJ), NCBI GenBank, NCBI Sequence Read Archive, or EMBL Nucleotide Sequence Database (ENA)). In your revised cover letter, please provide the relevant accession numbers that may be used to access these data. For a full list of recommended repositories, see [http://journals.plos.org/plosone/s/data-availability#loc-omics](http://journals.plos.org/plosone/s/data-availability#loc-omics) or <http://journals.plos.org/plosone/s/data-availability#loc-sequencing>.

We included all data generated during this study in the manuscript in supporting information S1 Table.

4. Please review your reference list to ensure that it is complete and correct. If you have cited papers that have been retracted, please include the rationale for doing so in the manuscript text, or remove these references and replace them with relevant current references. Any changes to the reference list should be mentioned in the rebuttal letter that accompanies your revised manuscript. If you need to cite a retracted article, indicate the article’s retracted status in the References list and also include a citation and full reference for the retraction notice.

We carefully revised our reference list and found no retracted manuscript. For some references we updated the pmid no. In page 17, Line 316 we replaced reference number 24 by number 53.

## Reviewers' comments:

Page 8, Line 35: delete has been and replace with was.

Page 10, Line 93: replace with were

Page 10, Line 96: Replace to with for

Page 11, Line 98: Delete an amount of and statement should read Briefly, 5ml of NaOH.......

Page 11, Line 113: Delete s

Page 11, Line 114-116: Rephrase statement not making sense M. bovis (	) and M. tuberculois (	) strains served as positive controls.

Page 12, Line 127: Replace by with using

Page 13, Line 150: Delete and replace with Out of

Page 13, Line 156: Remove, and add and

Page 15, Line 169: delete

Page 18, Line 223: Delete therefore

We changed the text in all sites of the manuscript as proposed by the reviewer.

Page 9, Line 65-67: This statement should be in the last paragraph at the end of the Introduction.

Page 9, Line 67-69: Added at the end of the introduction as part of the objectives description.

We moved both sentences to the end of the introduction paragraph.

Page 11, Line 106 : Berg et al. [15]

For reference no. 15 (Berg et al.) we added the doi of the Correction in the reference list.

Page 11, Line 120: Ruettger et al. [6]

For reference no. 6 (Ruettger et al.) it was not clear to us what the reviewer would have liked to changed, as this paper describes the used spoligotyping method in detail.
